# The genus *Nelcyndana* Stål (Hemiptera, Cicadidae, Taphurini) with description of three new species from Borneo

**DOI:** 10.3897/zookeys.61.487

**Published:** 2010-10-11

**Authors:** Joannes Petrus Duffels

**Affiliations:** Zoological Museum (Department of Entomology), University of Amsterdam, Plantage Middenlaan 64, NL-1018 DH Amsterdam, The Netherlands

**Keywords:** Nelcyndana, Taphurini, Cicadidae, taxonomy, new species, Borneo

## Abstract

The type species of Nelcyndana, Nelcyndana tener (Stål, 1870) from the Philippines, is redescribed and illustrated. The taxonomic position of the genus Nelcyndana in the tribe Taphurini is discussed. Three new species from Borneo are described and illustrated: Nelcyndana borneensis **sp. n.**, Nelcyndana vantoli **sp. n.**, and Nelcyndana mulu **sp. n.** Distributions maps for the three Borneo species are presented and a key for the identification of the four Asian species of the genus is provided.

## Introduction

The genus Nelcyndana was described by Stål (1870) as the new subgenus Nelcynda of the genus Tibicen Latreille, 1825. The new subgenus was erected for Nelcynda tener, a species from the Philippines also described by Stål in the same paper. In 1905, Distant elevated Nelcynda to genus rank and added a new species, Nelcynda madagascariensis, to the genus.The new name Nelcyndana was proposedby Distant (1906) since Nelcynda Stål proved to be preoccupied by Nelcynda Walker, 1862 used in Lepidoptera.

In his classic work “Cicadas of Malaysia”, Moulton (1923) assigned to Nelcynda tener the distinction of being the smallest cicada of the Malaysian region (Malay Peninsula, Sumatra, Borneo and Java). The expansion of tegmina of males and females of Nelcynda tener is 25–31 mm (Stål 1870), but Moulton (1923) recorded three even smaller specimens from Borneo with an expansion of 20–25 mm. The Borneo specimens differed from Nelcynda tener in the relative width of head and pronotum, but in spite of this, Moulton (l.c.) regarded these specimens inseparable from Nelcynda tener. Nelcyndana is separated from the other genera of the tribe Taphurini from Sundaland (Malayan Peninsula, Sumatra, Borneo and Java) by its small size and the four apical areas in the wings. Other genera of small cicadas in Sundaland of other tribes, like e.g., Muda Distant, 1897, have the normal number of six apical areas in the wings.

In the last two decades, cicada inventories in the Malaysian and Indonesian parts of Borneo and in Peninsular Malaysia have been strongly intensified. Zaidi and co-workers of Universiti Kebangsaan Malaysia recorded Nelcyndana tener from Sabah and Sarawak, Borneo (Zaidi and Ruslan 1998, Zaidi et al. 2000a, Zaidi et al. 2000b; Zaidi et al. 2004: 131–133, 137) and from Peninsular Malaysia (Zaidi and Ruslan 1997).

This study aims to contribute to a better taxonomic knowledge of the cicadas of Borneo anticipating a larger publication on the Bornean cicada fauna. The descriptions of the three new species of Nelcyndana from Borneo presented here are preceded by a discussion on the taxonomic position of the genus and a description of Nelcyndana tener from the Philippines, the type species of the genus. Several more undescribed species of Nelcyndana are awaiting description until more material comes available viz., three new species from Borneo, one from the Malay Peninsula and two from the Philippines.

## Material and methods

The institutions listed below are the depositories of the material studied. The abbreviations have been used in the lists of material and throughout the text.

BMNHNatural History Museum, London (former British Museum (Natural History))

NHRSNaturhistoriska Riksmuseet, Stockholm

NMWCNational Museum of Wales, Cardiff

RMNHNationaal Natuurhistorisch Museum (former Rijksmuseum voor Natuurlijke Historie), Leiden

ROMRoyal Ontario Museum, Toronto

ZMANZoölogisch Museum, Universiteit van Amsterdam, Amsterdam

Data on the distribution of the species were derived from the author’s “Biodiversity Database of the Cicadas of South East Asia and the West Pacific”, and plotted on maps of ADC-Worldmap version 2.0 vol. 4 Southern Asia & Australia with the program MapInfo for Power Mac, version 4.03. The localities and other data from the specimen labels in the database are filed in the program File-Maker Pro 4.0. The information about geographical co-ordinates has been retrieved from the following sources: “Atlas van Tropisch Nederland” (Anonymous 1938), “The Times Comprehensive Atlas of the World” (Anonymous 1999), and the GEOnet Names Server of the U.S. Defense Mapping Agency (http://www.nima.mil/gns/html/index.html).

The terminologyadopted in this paper for external features of the body and the male genitalia follows that of Duffels (Duffels 1977, 1983; Duffels and Turner 2002) and Moulds (2003, 2005).

## Taxonomy

The genus Nelcyndana was traditionally placed in the tribe Taphurini (Metcalf 1963; Duffels and Van der Laan 1985; Chou et al. 1997; Moulds 2005). Lee (2010) recently transferred Nelcyndana to the tribe Cicadettini. According to Lee (l.c.), Nelcyndana ‘..... is allied to Cicadetta Amyot, the type genus of the Cicadettini, considering the similarities in the male genitalia, especially the presence of a well-developed median lobe on the uncus’. Lee did not cite the publication of Moulds (2005) on the higher classification of cicadas. According to Moulds (l.c.) Cicadetiini can be separated from Taphurini by the presence of a pair of pseudoparameres branching off from the theca, the duck-bill shaped, very broad and flat uncus and the ventral rib of the aedeagus which is completely fused with the basal plate. In Taphurini the theca has no pseudoparameres, the uncus is absent and the ventral rib of the aedeagus is rod-like and suspended with attachments only at ends (Moulds 2005). The present study demonstrates that Nelcyndana has no thecal pseudoparameres but either one strong, chitinized appendage, apically divided in two stems, or a pair of more or less similar appendages, and a ventral rib of the aedeagus which is rod-like and suspended with attachments only at ends, which are both characters of the tribe Taphurini. Nelcyndana has no well-developed median uncus lobe as stated by Lee (2010). The uncus is absent, basal parts of the claspers extend to the basis of the anal lobe. The absence of the uncus is another character of the tribe Taphurini. This all means that Nelcyndana belongs to the tribe Taphurini.

### Nelcyndana

Distant, 1906

Tibicen (Nelcynda) Stål 1870: 716
Nelcynda Distant 1905: 35
Nelcyndana Distant 1906: 130 (nom. nov. pro Tibicen (Nelcynda) Stål [nec Nelcynda Walker 1862], Moulton 1923: 156, 157, 166; Metcalf 1963: 233–234; Duffels & Van der Laan 1985: 245; Chou et al. 1997: 80, 86; Moulds 2005: 393, 437; Lee 2010: 14, 26. [For further references before 1980 see: Metcalf 1963 and Duffels & Van der Laan 1985].

#### Type species:

Tibicen (Nelcynda) tener Stål 1870 by monotypy.

#### Diagnosis.

Small cicadas: body length male: 8.1–11.7 mm, female: 9.0–12.4 mm. Head slightly wider than pronotum collar. Vertex black, or reddish brown with a pair of paramedian, squarish, black markings or a pair of spots next to the paired ocelli. Postclypeus protruding weakly to fairly strongly with glabrous nose. Rostrum reaching beyond posterior margin of middle coxae or to anterior margin of hind coxae or beyond. Pronotum slightly wider than mesonotum. Mesonotum with a pair of paramedian, juxtaposed, black, obconical spots and a pair of black to black-brown lateral sigillae. Fore femora with four spines along lower ridge. Tegmina and wings hyaline, wings with four apical areas. Male operculum sickle-shaped and with adjacent setae, margins and apical third with long setae. Timbal with 6–7 ribs and faint intercalary ribs. Pygofer with about equitriangular dorsal beak with long and narrow apex; basal pygofer lobes very long reaching either to about apex of anal segment; upper pygofer lobes mostly distinctly separated from, but sometimes fused with, basal pygofer lobes. Uncus absent. Claspers juxtaposed and different in shape. Theca either with one strong, chitinized appendage, apically divided in two stems, or a pair of more or less similar appendages. Aedeagal basal plates in ventral view triangular to oval. Ventral rib of aedeagal basal plate rod-like and suspended with attachments only at ends.

#### Key to the males of Nelcyndana

**Table:** 

1.	Anterior and ventral parts of postclypeus yellowish to reddish brown. Philippines	Nelcyndana tener
–	Anterior and ventral parts of postclypeus black with either a yellowish to reddish brown glabrous nose or an oblong area of the same colour reaching from nose toward clypeal suture. Borneo	2
2.	Head width: 3.8–4.0 mm. Abdominal tergite 2 with a black transverse fascia along anterior margin, a reddish brown fascia at about half-length and a yellow-brown fascia along posterior margin. Borneo	Nelcyndana mulu
–	Head width: 2.8–3.3 mm. Abdominal tergite 2 black with a pair of paramedian yellow-brown spots at posterior margin. Borneo	3
3.	Male genitalia as in fig. 6	Nelcyndana borneensis
–	Male genitalia as in fig. 9	Nelcyndana vantoli

#### Nelcyndana tener

(Stål, 1870)

Figs 1–2

Tibicen (Nelcynda) tener Stål 1870: 716. Lectotype ♂: “Ins. / Philipp”, “Semper”, “Tibicen / tener / ♂ Stål”, “Typus” [printed in black cadre; red paper], “NHRS-HEMI 000000009” [examined].
Tibicen tener Distant 1890: Pl. vi, figs 5, 5a-b; Distant 1892: 130.
Nelcyndana tener Distant 1906: 139 (Equals Tibicen (Nelcynda) tener Stål); Moulton 1923: 157; Metcalf 1963: 234–235; Duffels & Van der Laan 1985: 245; Lee 2010: 14, 26 [For further references before 1980 see: Metcalf 1963 and Duffels & Van der Laan 1985]. Not: Nelcyndana tener; Zaidi and Ruslan 1997: 232[The specimen mentioned here from Pahang, Rompin probably belongs to a new undescribed species endemic to the Malayan Peninsula]; Zaidi and Ruslan 1998: 369; Zaidi et al. 2000b: 217; Zaidi et al. 2000a: 331; Zaidi et al. 2004: 131–133, 137. [The specimens mentioned in these publications come from Borneo (Sabah and Sarawak), and belong to one of the species from Borneo described here as new to science or to another new species.]

##### General.

The identity of Nelcyndana tener is established by the description given below. I have tried to find more specimens of Nelcyndana tener in various collections, but did not find one.

Nelcyndana tener can be distinguished from the other species of the genus from Borneo described here by the yellowish to reddish brown anterior and ventral parts of the postclypeus and the unpaired appendage of the theca. The Borneo species have a differently coloured postclypeus and a pair of more or less similar thecal appendages.

##### Lectotype designation for Nelcyndana tener (Stål).

Dr Gunvi Lindberg, curator of the Swedish Museum of Natural History, Stockholm, kindly sent me the type specimens of Nelcyndana tener for examination, viz., one male labelled holotype and two females labelled paratype. She also wrote me: …’ all curators seem to agree that the [type] labels are from the 20th century (thus not from Stål)’. In order to establish the identity of Nelcyndana tener, I designate here the male type specimen as the lectotype of Nelcyndana tener and the female type specimens as paralectotypes.

The lectotype is very fragile and partly damaged: the apical half of the wings is missing and the right tegmen is somewhat glued together; sternite 8 is partly damaged; the abdomen is glued to the head and thorax. The pygofer is taken out for the greater part. It is most likely that this damage to the lectotype was caused by an earlier student attempting to pull out the pygofer. The most characteristic feature of the genus Nelcyndana, viz., the four instead of five apical areas of the wing, already mentioned by Stål (1870) in his original description of the genus and the species, is not visible anymore in the lectotype.

The two paralectotypes are in good condition. One paralectotype bears the following labels: “Ins. / Philipp”, “Semper”, “Tibicen / tener Stål”, “Allotypus” [printed in black cadre; red paper], “NHRS-HEMI 000000010”. The other paralectotype bears the labels: “Ins. / Philipp”, “Semper”, “Tibicen / tener Stål / ♀”, “Paratypus” [printed in black cadre; red paper], “NHRS-HEMI 000000011”

**Figures 1–2.**
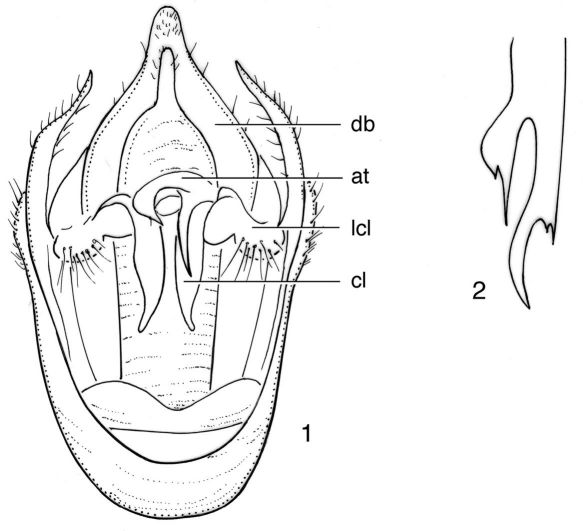
Nelcyndana tener, male lectotype. **1** pygofer in ventral view **2** appendage of theca. **at** appendage of theca; **cl** clasper; **db** dorsal beak; **lcl** lateral clasper lobe.

##### Description of male lectotype.

Ground colour reddish brown.

###### Head.

Vertex reddish brown with a pair of comma-shaped dark brown markings next to paired ocelli, a faint brownish ring around each of the ocelli, and dark brown colouration along mediodistal margin of eye. Postclypeus protruding weakly, yellowish to light reddish brown, dorsally with a pair of lateral dark brown spots, anterior and ventral parts of postclypeus with two paramedian series of 7 dark reddish brown transverse grooves. Anteclypeus light reddish brown without marking. Rostrum yellowish brown with brown apex reaching beyond posterior margin of middle coxae. Lorum black but anterior third reddish brown. Gena light reddish brown with a black line along posterior eye margin. Antenna, supra-antennal plate and vertex lobe yellowish brown to reddish brown.

###### Thorax.

Pronotum with a pair of large, rounded rectangular, dark reddish brown to black brown markings that are enclosed by the reddish brown anterior margin of pronotum, the light reddish brown pronotum collar, and a broad median, light reddish brown fascia that strongly widens to anterior margin of pronotum and to pronotum collar. A lanceolate black-brown marking connects the broadly black-brown anterior and posterior oblique fissures.

###### Mesonotum

with a pair of paramedian, black-brown, obconical spots, those are fused at anterior margin of mesonotum and reach to one fourth of mesonotum disk. Scutal depressions in front of cruciform elevation with light brownish suffusion. Lateral sigillae clouded with dark brown and anteriorly slightly wider than anterior part of paramedian obconical spots, gradually narrow to their distal ends near anterior angles of cruciform elevation. Cruciform elevation yellowish.

###### Legs.

Yellow-brown to brownish. Fore femora with four yellow-brown spines with brown apices: a long spine at proximal end of lower ridge of femur, a second spine, half as long as proximal spine, at half-length of lower ridge, a third spine, one third as long as proximal spine, at three fifths of lower ridge, and a tiny spine near distal end of lower ridge.

###### Tegmina and wings.

Hyaline. Venation of tegmina and wings brownish to reddish brown variegated with dark brown.

###### Operculum.

Sickle-shaped with narrowly rounded apex reaching to almost anterior margin of abdominal segment 2, strongly narrowed from base to one fourth of its length, and gradually narrowing from one fourth of length to apex. Apical half with sparse long setae, especially along operculum margins.

###### Abdomen.

Timbal with 7 evenly spaced long ribs and very faint intercalary ribs. Tergite 1 dark reddish brown, tergites 2–7 with a laterally widening, dark reddish brown fascia along anterior margin, medially reaching to half-length or two thirds of tergite, and a laterally narrowing reddish brown fascia; tergite 3 also with a narrow yellowish fascia along posterior margin. Tergite 8 with a laterally narrowing dark reddish brown fascia along anterior segment margin and a laterally narrowing reddish brown fascia, both two fifths as long as tergite and a yellowish fascia along posterior margin, one fifth as high as the tergite. Sternite 2 yellowish, sternites 3 to 6 and anterior half of sternite 7 reddish brown, posterior half of sternite 7 and whole sternite 8 yellowish.

###### Genitalia

(Figs 1–2). Pygofer with convex lateral sides. Dorsal beak about equitriangular, brownish and with somewhat darker brown, fairly long and narrow apex. Basal pygofer lobe long and weakly convex, narrowed to acute apex, that is incurved and reaches to half-length of anal segment. Upper pygofer lobe very short, rounded and widely separated from basal pygofer lobe. Claspers juxtaposed, fairly narrow, incurved; lateral margin distinctly concave at base and weakly convex to narrow and slightly outcurved apex; medial margin very weakly convex; lateral clasper lobes protruding and spherical. Theca with one strongly chitinized appendage (Fig. 2), which is apically divided in a long, curved, dagger-shaped stem with a strong spine at base and a shorter, more widened, dagger ending in an acute spine. Aedeagal basal plates in ventral view triangular.

##### Description of female paralectotypes.

There are two female paralectotypes, one fully coloured and one with obsolete marking. A description of the fully coloured female follows here:

###### Head.

As in male lectotype but anteclypeus black-brown with light brownish anterior margin and keel, and vertex with additional dark brown marking between paired ocelli and eyes.

###### Thorax.

Pronotum as in male lectotype. Mesonotum as in male lectotype but paramedian obconical spots reaching to one third of mesonotum disk and lateral sigillae black-brown.

###### Legs, tegmina and wings.

As in male lectotype.

###### Operculum.

Basal half broad, narrowed at half its length to two thirds of basal width; apical part curved mediad with narrowly rounded apex reaching to just beyond anterior margin of sternite 2.

###### Abdomen.

Tergite 1 light reddish brown. Tergite 2 reddish brown with laterally narrowing, black fascia along anterior margin medially reaching to two fifths of segment length. Tergites 3–7 with a laterally widening, black fascia along anterior margin, medially reaching to half or three fifths of segment length, a slightly narrower reddish brown fascia at about half-length of tergite and a narrow yellowish green fascia along posterior margin. Tergite 8 with laterally narrowing, black fascia along anterior margin medially reaching to one fourth of segment length, a broad reddish brown fascia and a fairly narrow, yellowish green fascia along posterior margin. Sternite 2 with dark brown transverse marking, sternites 3 to 6 with dark brown transverse band, which is a little less than half as wide as sternite and reaches from anterior sternite margin to two thirds or three fourths of sternite length. Sternite 7 medially dark brown. Segment 9 dorsally with a pair of oblong, paramedian, black-brown markings reaching from anterior margin of segment to three fourths of its length, and laterally with a pair of, round, black-brown spots.

The female paralectotype with the more obsolete marking has no marking on anteclypeus, no additional brown marking on vertex, very light brown lateral sigillae on mesonotum, light reddish brown abdominal tergites with much narrower black marking along their anterior margins, and only small brown median spots on sternites 5 and 6.

###### Measurements

(in mm; 1♂, 2♀). –Body length ♂: 10.2 ♀: 11.5; tegmen length ♂: 10.9, ♀: 12.9–13.5; head width ♂: 3.2, ♀: 3.7–3.8; pronotum width ♂: 3.1, ♀: 3.6–3.7.

###### Distribution.

The type specimens of Nelcyndana tener bear a label with the unspecified locality “Ins. / Philipp”. I have tried to find more specimens of this species in various collections, but did not find one. Lee (2010) recorded Nelcyndana tener from Mindanao, Philippines, but this record needs confirmation since several undescribed species of Nelcyndana occur in the Philippines.

#### Nelcyndana borneensis

Duffels sp. n.

urn:lsid:zoobank.org:act:EF857435-0490-41F5-B69C-C8A62CA07F93

Figs 3 5 7 

##### Type material.

24♂ 23♀. **Holotype** ♂: **Malaysia: Sabah:** “RMNH Leiden E SABAH / Lahad Datu, 60 km W of: / Danum Valley Field Centre / at junction Sg Segama and / Sg Palum Tambun, 150 m / 4°58"N 117°48'E”, “At light. Bridge of Segama. / 19 Mar 1987, 18.30–21.30. / Clearing, edge of untouched / evergr. lowl. rainforest / leg. Van Tol & Huisman” (RMNH). **Paratypes: Malaysia: Sabah:** same data as holotype, 3♀ (RMNH), same data as holotype but: 17 & 18.iii.1987, 18.30–21.00, 1♂ 2♀ (RMNH), 20.iii.1987, 18.20–21.00, 1♂ 2♀ (RMNH), clearing nr E trail, 21.ii.1987, 18.30–20.30, 1♀ (RMNH); 60 km W Lahad Datu, DVFC, nr Segama bridge, 4°58'N 117°43'E, 20.x.1987, 150 m, J. Huisman & R. de Jong, 1♂ (RMNH); 60 km W of Lahad Datu, road Kg Silam – DVFC, km 68.5, 4°58'N 117°48'E, 150 m, 24.iii.1987, ML-light, J. Huisman, 1♂ (RMNH); Danum Valley, 5°01'N 117°47'E, 10.ix.1987, 100 m, A.H. Kirk-Spriggs, nmw Sabah (Borneo) Expedition, nmw. z 1987, 094, light trap sample roadside, secondary forest, 2♂ (NMWC), same data but: 11.ix.1987, 1♂ 2♀ (nmwc), 14.x.1987, 1♀ (NMWC); Danum Valley, 5°01'N 117°47'E, 30.ix.1987, 200 m, A.H. Kirk-Spriggs, nmw Sabah (Borneo) Expedition, nmw., z 1987, 094, lowland mixed dipterocarp forest, Grid EZ, light trap sample, understory forest, 1♂ 2♀ (nmwc); Danum Valley, 70 km W Lahad Datu, Field Centre, Main Trail West 0 North 5, 150 m, 15.xii.1989, sample Sab. 69, secondary vegetation/canopy/primary forest margin, at light, M.J. & J.P. Duffels, 1♂ (ZMAN); Bettotan, nr. Sandakan, 17.viii.1927, C.B.K. & H.M.P. F.M.S. Museums, ex. F.M.S. Museum, B.M. 1955–354, 1♂ (BMNH), same data but 24.vii.1927, 1♂ (BMNH); Sungai Darling, 60 m W Sandakan, 26.xi.1989, sample Sab. 43, secondary forest understorey, at light, M.J. & J.P. Duffels, 1♀ (ZMAN). **Sarawak:** Foot of Mt. Dullit, junction of rivers Tinjar & Lejok, 25.viii.1932, Light trap, Oxford Univ. Exp. B.M. Hobby & A.W. Moore B.M. 1933–254, 2♂ 3♀ (BMNH), same data but 28.viii.1932, 1♀ (BMNH), 31.viii.1932, 1♂ 1♀ (BMNH), 6.x.1932, 1♂ (BMNH); Gunung Mulu Nat. Park, Site 7, Long Pala (Base), 324450, 50 m, Alluvial/secondary forest, Acl-understorey, J.D. Holloway, RGS Mulu exped., B.M. 1978–206, 4♂ 4♀ (BMNH). **Brunei:** Temburong District, ridge NE of Kuala Belalong, approx. 300 m, x.1992, J.H. Martin, 125 W mv light, B.M. 1992–172, 3♂ (BMNH), same data but: xi.1992, 1♂ (BMNH).

**Figures 3–4.**
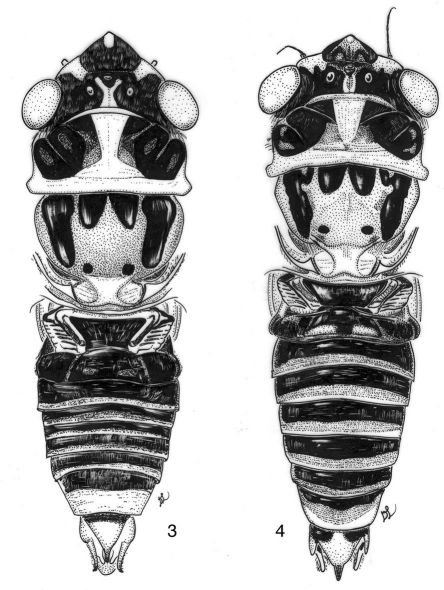
Nelcyndana spec., male body in dorsal view. **3** Nelcyndana borneensis, paratype, Sarawak, Gunung Mulu, site 7 **4** Nelcyndana vantoli, holotype.

##### Etymology.

This species name refers to its distribution in Borneo.

##### Description.

Ground colour yellowish to greenish brown, and reddish brown. Marking black or black variegated with reddish brown. Dorsal side of body silvery pilose, ventral side with longer silvery setae.

**Figures 5–6.**
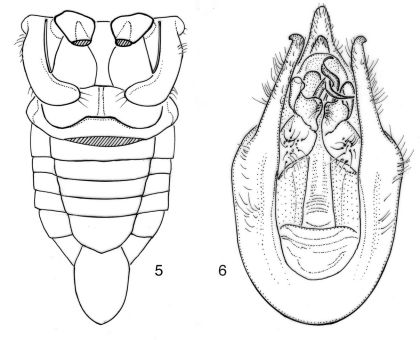
Nelcyndana borneensis. **5** male abdomen in ventral view, paratype, Sarawak, Gunung Mulu, site 7 **6** male pygofer in ventral view, paratype, Sabah, Danum Valley Field Centre.

###### Male.

####### Head

(Fig. 3). Vertex black, with exception of a yellow area adjacent to supra-antennal plate and a yellow median triangle against posterior margin of head; in a few specimens the yellow colouration is more extended, leaving only the lateroproximal parts of the vertex black. Postclypeus protruding fairly strongly, black or black variegated with reddish brown, but anteriorly with greenish, glabrous nose, ventrally sometimes with reddish brown oblong marking from nose towards clypeal suture; anterior and ventral parts with 6 pairs of distinct, black transverse ridges, lateral margins of ventral part yellowish brown. Anteclypeus black, often with either basal triangle on medial keel or whole keel reddish brown. Rostrum brown with dark brown apical part reaching beyond anterior margin of hind coxae. Lorum and lateroproximal part of gena black. Antennae, supra-antennal plate and vertex lobe yellowish brown.

####### Thorax.

Pronotum (Fig. 3). A pair of large, rounded rectangular, black markings, sometimes slightly variegated with reddish brown, is enclosed by a narrow, yellowish to greenish fascia along anterior pronotal margin, the pronotum collar of the same colour, and a broad median fascia of the same colour that strongly widens to the anterior margin of the pronotum and to the pronotum collar.

####### Mesonotum

(Fig. 3) with a pair of paramedian, black, obconical spots at anterior margin, reaching to one fifth to one third of mesonotum disk; spots either juxtaposed or fused at base. Scutal depressions in front of cruciform elevation covered by often fairly large round, black spots. Lateral sigillae black, anteriorly 1.5 times as broad as anterior part of paramedian obconical spots, narrow a little abruptly at one fourth of its length from base and then gradually narrow to their distal ends. Cruciform elevation yellowish.

####### Legs.

Yellow-brown, fore and middle tarsi and distal part of fore tibia often darker brown. Fore femora with four yellow-brown spines with dark brown apical parts: a long, erect spine at proximal end of lower ridge of femur, a second spine, two-thirds as long as proximal spine, at three-fifths of lower ridge, a third, slightly shorter, spine, at four-fifths of lower ridge and a very short, triangular spine near distal end of lower ridge.

####### Tegmina and wings.

Hyaline. Venation of tegmina and wings yellowish to reddish brown.

####### Operculum

(Fig. 5). Sickle-shaped with rounded apex reaching to either two-thirds or three-fourths of timbal cavity or to almost anterior margin of abdominal segment 2, strongly narrowed from base to one fourth of its length, about equally wide in apical three fourths. Surface of operculum with short to fairly long, adjacent setae and apical one third with very long setae especially along margins of operculum.

####### Abdomen

(Figs 3, 5). Timbal with 6 somewhat irregular evenly spaced long ribs and very faint intercalary ribs. Tergite 1 black or black-brown, tergite 2 black with a pair of paramedian yellow-brown spots at posterior margin, tergites 3–8 with a laterally widening, black fascia along anterior margin medially reaching to one third or two thirds of segment length, a laterally narrowing reddish brown fascia at about half-length of tergite and a laterally narrowing yellowish green fascia along posterior margin. Sternite 2 brownish black, sternites 3 to 7 black to black-brown but sternites 3 to 6 yellowish along posterior margin, sternite 8 castaneous.

####### Genitalia

(Fig. 6). Pygofer with more or less parallel lateral sides. Dorsal beak fairly long, slightly upcurved, yellowish brown, with black apex. Basal pygofer lobe very long, straight and narrow, apically outcurved and reaching about apex of anal segment. Upper pygofer lobes in ventral view hidden behind the basal lobes adjacent to basal pygofer lobe, narrowly rounded apically and about one fifth as long as apical part of basal lobe measured from base of upper pygofer lobe to its apex.Dorsal beak in dorsal view about an equilateral triangle with nipple-shapedapex. Clasper with basal two thirds about oval, and apical one third with a quadrangular median angle and an apically rounded lateral flap with a short spine at ventral margin. Theca chitinized, apically with a pair of long and slender, apically acute and curved, appendages. Aedeagal basal plates in ventral view triangular with strongly elongated anterior angles.

###### Female.

####### Head

as in male, but postclypeus reddish brown from nose to anterior margin of pronotum; vertex reddish brown with exception of the black lateroproximal parts that more or less enclose the paired ocelli; a black spot is attached to proximal side of median ocellus.

####### Thorax. Pronotum.

Rectangular markings as described for males are not black but reddish brown with broad black marking in the oblique fissures and narrow black marking in lateral part of ambient fissure. Mesonotum as in males.

####### Legs, tegmina and wings

as in males.

####### Operculum.

Basal half broad, narrowed at half its length to two thirds of basal width; apical part curved mediad, medial margin weakly concave, and lateral margin convex to narrowly rounded apex, reaching to anterior margin or one third of sternite 2.

####### Abdomen.

Tergites 2–7 with a laterally widening, black fascia along anterior margin medially reaching to one third or two thirds of segment length, a laterally narrowing, reddish brown fascia at about half-length of tergite and a laterally narrowing yellowish, green fascia along posterior margin. Tergite 8 with narrow black fascia and broader reddish brown and yellowish fasciae. Sternite 2 medially black-brown, sternites 3 to 6 with brown-black transverse band, which is one third to half as wide as sternite and reaches from anterior sternite margin to two-thirds of sternite or at most almost to its posterior margin. Segment 9 with a pair of oblong, paramedian, brown to black-brown markings and a pair of round, lateral, brown spots and brownish colouration along basal two-thirds of lower margin.

##### Measurements

(in mm; 6♂, 6♀). Body length ♂: 8.1–9.2 ♀: 9.0–10.3; tegmen length ♂: 8.7–10.2, ♀: 10.0–11.8; head width ♂: 2.8–3.2, ♀: 2.9–3.7; pronotum width ♂: 2.5–2.9, ♀: 2.8–3.4.

##### Distribution

**Figure 7.**
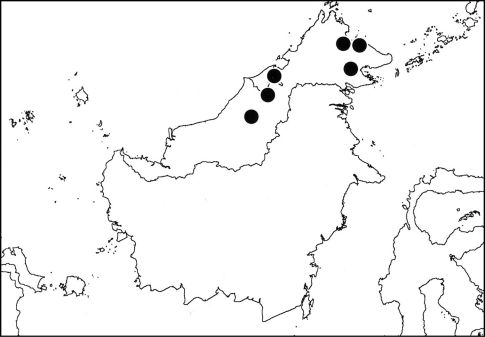
Distribution of Nelcyndana borneensis.

(Fig. 7). Nelcyndana borneensis is known from various places in Borneo (Sarawak, Sabah, Kalimantan and Brunei) and is probably a Borneo endemic.

#### Nelcyndana vantoli

Duffels sp. n.

urn:lsid:zoobank.org:act:0C398A2C-6009-4673-B3BF-49F072AEBB64

Figs 4 8 10 

##### Type material.

2♂. **Holotype** ♂: **Malaysia: Sabah:** “RMNH Leiden E SABAH / Lahad Datu, 60 km W of: / Danum Valley Field Centre / at junction Sg Segama and / Sg Palum Tambun, 150 m / 4°58"N 117°48'E”, “At light. Bridge of Segama. / 26 Mar 1987, 18.30–21.30. / Clearing, edge of untouched / evergr. lowl. rainforest / leg. Van Tol & Huisman” (RMNH). **Paratype: Indonesia: Kalimantan Timur:** Long Tua, edge of Bahau River, 3°10'N 115°47'E, 440 m, 5–9.iv.1994, UV light, B. Hubley & D.C. Darling, IIS 940526, 1♂ (ROM).

**Figure 8–9.**
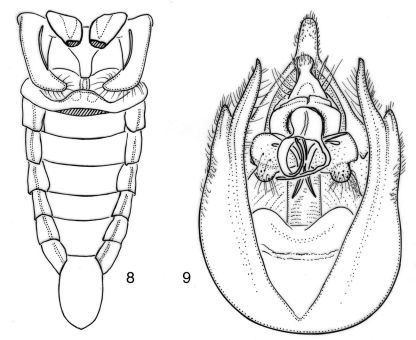
Nelcyndana vantoli, holotype. **8** male abdomen in ventral view **9** male pygofer in ventral view.

##### Etymology.

This species is dedicated to my colleague and friend Dr Jan Tol, odonatologist of the Leiden museum, for his significant contribution to our knowledge of the cicadas of Borneo.

##### Description of male holotype.

Ground colour yellowish to reddish brown. Marking black or black variegated with reddish brown. Ventral side of body with short silvery setae.

###### Head.

(Fig. 4). Vertex reddish brown with a pair of paramedian, squarish, black markings, separated by a median reddish brown triangle at posterior margin of head and reaching from posterior margin of head to two thirds of vertex length beyond the paired ocelli. Frons black-brown. Postclypeus protruding fairly weakly, black variegated with reddish brown, medial part reddish brown from its glabrous nose ventrally to clypeal suture and dorsally to frontoclypeal suture; anterior and ventral parts of postclypeus black with 6 pairs of distinct, black transverse ridges, lateral margins of ventral part yellowish brown. Anteclypeus medially reddish brown, with dark brown mark at two thirds of keel, and turning to dark brown laterad. Rostrum brown with dark brown apical part reaching anterior margin of hind coxae. Lorum black with exception of brownish anterior angle. Gena black but brownish around antenna. Antennae, supra-antennal plate and vertex lobe yellowish to reddish brown.

###### Thorax.

Pronotum (Fig. 4). A pair of large, rounded rectangular, black markings, sometimes slightly variegated with reddish brown, is enclosed by a narrow, yellowish to greenish fascia along anterior pronotal margin, the pronotum collar of the same colour, and a broad median fascia of the same colour that strongly widens to the anterior margin of the pronotum and to the pronotum collar.

###### Mesonotum

(Fig. 4) with a pair of paramedian, juxtaposed, black, obconical spots at anterior margin, reaching to one fourth of mesonotum disk. Scutal depressions in front of cruciform elevation covered with small round, brown spots. Lateral sigillae mainly black, anteriorly 1.5 times as broad as anterior part of paramedian obconical spots, gradually narrow from base to distal end; basomedial part of sigillae variegated with the ground colour. Cruciform elevation yellowish.

###### Legs.

Yellow-brown to greenish. Fore femora with four reddish brown spines: a long spine at proximal end of lower ridge, a second spine, two-thirds as long as proximal spine, at two thirds of lower ridge, a third spine, half as long as proximal spine at four fifths and a very short, triangular spine near distal end of lower ridge.

###### Tegmina and wings.

Hyaline. Costa and basal half of radius + subcosta of tegmen reddish brown, radius anterior and distal half of subcosta reddish to dark brown. Remaining venation of tegmina and wings light to dark brown.

###### Operculum.

(Fig. 8). Sickle-shaped with rounded apex reaching to three-fourths of timbal cavity, strongly narrowed from base to one fourth of its length, about equally wide in apical three fourths. Surface of operculum with sparse, adjacent setae and apical one third with very long setae especially along margins of operculum.

###### Abdomen.

(Figs 4, 8). Timbal with 6 somewhat irregular evenly spaced long ribs and faint intercalary ribs. Tergite 1 black-brown, tergite 2 dark brown to black with a pair of paramedian, transverse, reddish brown markings close to posterior margin, tergites 3–8 with a laterally widening, dark brown to black fascia along anterior margin medially reaching to half or two thirds of segment length, a laterally narrowing reddish brown fascia at about half-length of tergite and a narrow yellowish fascia along posterior margin. Sternite 2 brownish black, sternites 3–5 brownish black from anterior sternite margin to about three fourths of sternite length but with lateral reddish stripes, posterior margin yellowish; sternites 6–7 brownish black, sternite 8 light brown.

###### Genitalia.

(Fig. 9). Pygofer with convex lateral sides. Dorsal beak about equitriangular, reddish, with long and narrow, brown apex. Basal pygofer lobe long and convex, and reaching just beyond apex of anal segment. Upper pygofer lobe fused with basal pygofer lobe, narrowly rounded apically and about one fifth as long as apical part of basal lobe measured from base of upper pygofer lobe to its apex.Clasper basally round and flat with a medial, long and narrow, slightly flattened, incurved spine, a shorter slightly curved lateral spine and a round protrusionat its proximal margin; medial spines of both claspers juxtaposed and of unequal length. Theca chitinized, apically with a long and fairly slender, curved chitinized appendage, and a somewhat shorter and thinner appendage; the broadest appendage with very thin spine arising from its base. Aedeagal basal plates in ventral view divided in rounded, oval lobes.

##### Measurements

(in mm; 2♂). Body length 10.4–10.7; tegmen length 10.8–12.0; head width 3.2–3.3; pronotum width 2.9–3.1.

##### Remark on paratype.

The paratype from Kalimantan Timur is alike to the holotype in the male genitalia, but the marking on the body is more extended and black instead of black-brown. The head is black with exception of the medial triangle at posterior margin of head, the nose of the postclypeus and the supra-antennal plates, which are reddish brown. The pronotum has a pair of black squarish markings, the mesonotum is entirely black, and the abdomen has a similar marking as the holotype, but the marking is black instead of brownish black.

##### Distribution

**Figure 10.**
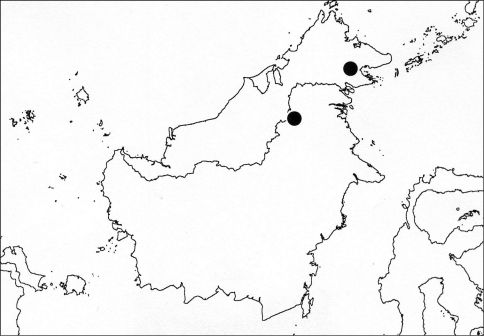
Distribution of Nelcyndana vantoli.

(Fig. 10). Nelcyndana vantoli seems to have a restricted range in northeastern Borneo. It is known from a specimen from Danum Valley N.P. in northeastern Sabah and a specimen from Long Tua in the northeastern part of Kalimantan Timur.

#### Nelcyndana mulu

Duffels sp. n.

urn:lsid:zoobank.org:act:405FB5BF-13CE-4242-BC2B-CA1060A4CDC6

Figs 11 14 

##### Type material.

**Holotype** ♂: **Malaysia, Borneo: Sarawak:** “SARAWAK / Gunong Mulu / Nat. Park”, “Site 23, April / W. Melinau Gorge / 250 m. 430558”, “J.D. Holloway / RGS Mulu exped. / B.M. 1978–206”, “FEG 4. Limestone / forest. MV - / canopy/understorey”(BMNH). **Paratypes: Malaysia, Borneo, Sarawak:** same data as holotype, 4♂ 3♀ (BMNH).

**Figure 11.**
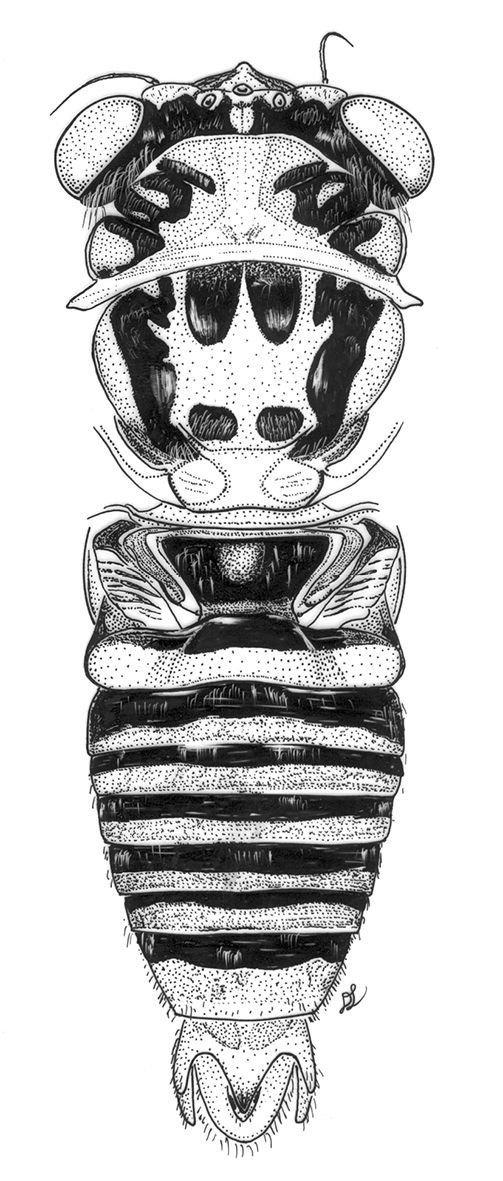
Nelcyndana mulu, holotype, body in dorsal view.

##### Etymology.

This species name refers to its occurrence in Gunung Mulu National Park, Sarawak, Borneo.

##### Diagnosis.

Nelcyndana mulu is distinctly larger than Nelcyndana borneensis and Nelcyndana vantoli (head width ♂: Nelcyndana mulu: 3.8–4.0 mm; Nelcyndana borneensis: 2.8–3.2 mm and Nelcyndana vantoli 3.2–3.3 mm).The males of Nelcyndana mulu can also be separated from the other two species by the colour of the male abdominal segment 2. The anterior one third of male abdominal segment 2 is black in Nelcyndana mulu, while the whole segment is black in Nelcyndana borneensis and Nelcyndana vantoli.

##### Description.

Ground colour and pilosity as in Nelcyndana borneensis.

###### Male.

####### Head

(Fig. 11). Vertex reddish brown with exception of a pair of paramedian, squarish, black markings between paired ocelli and posterior margin of head. Postclypeus fairly weakly protruding, medially yellowish to light reddish brown from its glabrous nose to frontoclypeal suture and ventrally (almost) to clypeal suture; lateral parts of postclypeus dark reddish brown to blackish with 6 pairs of distinct, black transverse ridges, grooves between ridges sometimes reddish brown, lateral margins of ventral part yellowish brown. Anteclypeus black with reddish brown basal triangle. Rostrum brown with dark brown apical part reaching beyond anterior margin of hind coxae. Posterior two thirds of lorum black, anterior part reddish brown. Gena reddish brown but black along inner margin of eye. Antennae, supra-antennal plate and vertex lobe yellowish to reddish brown.

####### Thorax.

Pronotum (Fig. 11). A pair of large, rounded rectangular, reddish brown markings enclose a variable pattern of black marking; the reddish brown rectangles are enclosed by a narrow, greenish to yellow-brown, fascia along anterior pronotal margin, the pronotum collar of the same colour, and a broad median fascia of the same colour that strongly widens to anterior margin of pronotum and to pronotum collar.

####### Mesonotum

(Fig. 11) with a pair of paramedian, black, obconical spots at anterior margin, reaching to one third to two fifths of mesonotum disk. Scutal depressions in front of cruciform elevation covered by round, black spots. Lateral sigillae black to black-brown or clouded with black to black-brown, anteriorly 1.5 times as broad as anterior part of paramedian fasciae, narrow a little abruptly at one fourth of its length from base and gradually narrow to their distal ends. Cruciform elevation yellowish.

####### Legs.

Yellow-brown, fore tarsi and fore tibia darker brown; inner side of fore femur with dark brown marking. Fore femora with four, fairly stout, black-brown spines with light brown apices: a long and strong spine at proximal end of lower ridge of femur, a distinctly shorter spine at half-length of lower ridge, a somewhat shorter spine at four fifths of lower ridge and a very short, triangular spine near distal end of lower ridge.

####### Tegmina and wings.

Hyaline. Venation of tegmina and wings greenish to reddish brown.

####### Operculum

**Figures 12–13.**
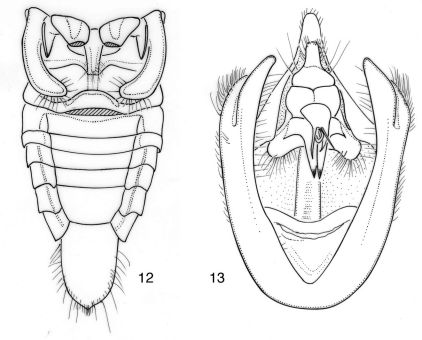
Nelcyndana mulu, holotype **12** male abdomen in ventral view **13** male pygofer in ventral view.

(Fig. 12). As in Nelcyndana borneensis, reaching to three-fourths of timbal cavity or to almost anterior margin of abdominal segment 2,

####### Abdomen

(Figs 11–12). Timbal with 6 evenly spaced long ribs and very narrow and faint intercalary ribs. Tergite 1 black or black-brown, tergites 2–8 with a laterally slightly widening, black fascia along anterior margin medially reaching to one third of segment or half-length, a laterally slightly narrowing reddish brown fascia at about half-length of tergite and a yellow-brown fascia along posterior margin. Sternites 2 to 7 for the greater part black-brown but yellow-brown along posterior margins; sternite 8 yellow-brown.

####### Genitalia

(Fig. 13). Pygofer with convex lateral sides. Dorsal beak about equitriangular, slightly upcurved and yellowish brown with long and narrow, black-brown apex. Basal pygofer lobe long and convex, apically flattened and reaching about apex of anal segment. Upper pygofer lobe narrowly separated from basal pygofer lobe and two thirds as long as apical part of basal lobe measured from base of upper pygofer lobe to its apex. Claspers juxtaposed, narrow, incurved and narrowing to acute apex and with laterobasal equilateral triangular, protrusions; lateral clasper lobes protruding and rounded. Aedeagus apically slightly upcurved. Apex of theca with two fairly narrow dagger-shaped appendages, and a very thin, long spine and a very thin, short spine. Aedeagal basal plates triangular.

###### Female.

####### Head.

Vertex reddish brown with a black spot laterally of paired ocelli only or with black marking restricted to lateroproximal part of vertex. Postclypeus reddish brown to black, but medially yellowish from nose to frontoclypeal suture or from frontoclypeal suture to (almost) clypeal suture; lateral parts of postclypeus with 7 pairs of brown to black transverse ridges. Anteclypeus and rostrum as in male. Antenna, supra-antennal plate, vertex lobe and gena yellowish to reddish brown. Lorum reddish brown but posterior two thirds of lorum more or less black in two paratypes.

####### Thorax.

Pronotum. As in male, but the rectangular, reddish brown markings do not enclose any black marking.

####### Mesonotum

with a pair of paramedian, juxtaposed, black to brown, obconical spots at anterior margin, reaching to two fifths of mesonotum disk. Scutal depressions in front of cruciform elevation with round, brown spots. Lateral sigillae black to very faint and reddish brown without black marking. Cruciform elevation yellowish.

####### Legs, tegmina and wings

as in male

####### Operculum.

Basal half broad, operculum narrowed at half its length to two thirds of basal width, medial margin concave, lateral margin of apical part basally parallel to medial margin, laterodistal angle obtusely rounded, apical margin straight, reaching to anterior margin or one third of sternite 2 and making an angle of 60 degrees with medial margin.

####### Abdomen.

One paratype has the following marking: tergite 2 with black-brown fascia, about as broad as cruciform elevation, along anterior tergite margin, tergites 3–7 with transverse, black fasciae along anterior margins, that on tergite 3 reaches medially to two fifths of tergite length, that on tergite 4 to one third and those on tergites 5–8 to one fourth or one fifth of tergite length, the fasciae on tergites 3–8 widen laterally to reach to two thirds of tergite length. Sternite 2 medially black-brown, sternites 3 to 6 with brown-black transverse band, which is two thirds as broad as sternite and reaches from anterior sternite margin to two-thirds of sternite, sternite 7 medially black-brown. Segment 9 dorsally with a pair of oblong, paramedian, dark brown markings, laterally with a pair of round, lateral, brown spots and ventrally with brownish colouration along basal two-thirds of lower margin. The other two paratypes differ in the following features: dorsal marking with much narrower and partly missing fasciae; sternite 2 medially brownish, sternite 3 with black-brown transverse band, half as wide as sternite, sternites 4–6 with dark brownish median marking, one third to half as wide as sternite. Segment 9 similar but missing the lateral spots.

##### Measurements

(in mm; 5♂, 2♀). Body length ♂: 9.9–11.7, ♀: 12.0–12.4; tegmen length ♂: 12.7–12.9, ♀: 14.2–15.0; head width ♂: 3.8–4.0, ♀: 4.0–4.3; pronotum width ♂: 3.6–3.8, ♀: 3.8–4.1.

##### Distribution

**Figure 14.**
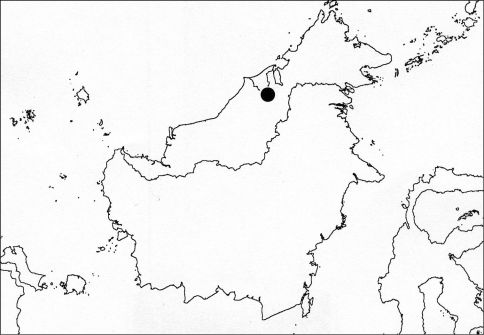
Distribution of Nelcyndana mulu.

(Fig. 14). Nelcyndana mulu is only known from the Melinau Gorge in Gunung Mulu N.P. in Sarawak).

## Supplementary Material

XML Treatment for Nelcyndana
